# Supplementary material for: Integrating 4 methods to evaluate physical function in patients with cancer (In4M): protocol for a prospective cohort study
Source: BMJ Open. 2024 Jan 10;14(1):e074030. doi: 10.1136/bmjopen-2023-074030 (PMC10806877; doi:10.1136/bmjopen-2023-074030)
Supplement: Supplementary data [file bmjopen-2023-074030supp001.pdf]

Appendix (Supplementary Material) 1: Inclusion and Exclusion Criteria for the In4M Study

1.1.1. Inclusion Criteria

1) Age 18 and over;
2) English- or Spanish-speaking;
3) Pregnant and non-pregnant patients are eligible for participation in this study
4) Eligible cancer type and planned intravenous cytotoxic chemotherapy regimen (defined as including 1 or more cytotoxic agents)
5) ECOG Performance Score of < 3
6) Breast cancer patients
   a) Patients with any stage breast cancer for whom a new intravenous cytotoxic chemotherapy regimen is planned within the next 8 weeks (patients with local/regional/distant recurrences are allowed; patients with concurrent/prior/future immunotherapy/radiotherapy, targeted therapy, and endocrine therapy for breast cancer are allowed)
7) Lymphoma patients
   a) Lymphoma patients of any histology, stage or line of treatment planned to receive a new intravenous cytotoxic containing chemotherapy regimen (patients planned to receive radiation, maintenance chemotherapy, consolidation stem cell transplant or chimeric antigen receptor T (CAR-T) cell therapy are allowed)
8) If patients are receiving the above standard therapies as part of a clinical trial which may include a novel agent or combination, they are also eligible for the present study if the therapeutic protocol permits enrollment in both studies
9) Willing and able to give consent and participate in study
10) Able to access a mobile smartphone or tablet or computer with web access every day to complete study surveys; able to regularly upload data from the Fitbit to a device in a way that it can be transferred to Hugo.
11) Willing and able to perform an in-clinic 6-minute walk test (gait aides are permitted if regularly used by the patient). If a patient is recruited remotely outside of Mayo Clinic Rochester or Yale Smilow Cancer Center New Haven, 6-minute walk test may be omitted.
12) Willing to use the health data sharing platform

Potential subjects who do not meet all of the enrollment criteria will not be enrolled. Any deviations from these criteria must be reported in accordance with IRB Policies and Procedures.

1.1.2. Exclusion Criteria

1) Prior intravenous cytotoxic chemotherapy within 3 weeks prior to study enrollment

2) Excluded regimens (due to length of hospitalization required for chemotherapy administration):

   a) R-CODOX-M/IVAC,
   b) DA-R-EPOCH (inpatient)

3) Excluded histology (due to length of hospitalization and inpatient predominant treatment for required chemotherapy): primary central nervous system lymphoma

   a) Other regimens with an anticipated high duration of inpatient care time, at PI discretion

4) Lack of access to a mobile smartphone or tablet or computer with web access

5) Unable or unwilling to upload data from the Fitbit

6) Unable or unwilling to use the health data sharing platform

7) Unable to give consent and be enrolled
