# Supplementary material for: Integrating 4 methods to evaluate physical function in patients with cancer (In4M): protocol for a prospective cohort study
Source: BMJ Open. 2024 Jan 10;14(1):e074030. doi: 10.1136/bmjopen-2023-074030 (PMC10806877; doi:10.1136/bmjopen-2023-074030)
Supplement: Supplementary data [file bmjopen-2023-074030supp003.pdf]

Supplementary Table 1: Schedule of Assessments

			Standard 2-6 month intravenous chemotherapy treatment; total 9 months study follow up												
	**Lead-in**	**BL**	**W2**	**W3**	**W4**	**W5**	**W6**	**W7**	**W8**	**M3**	**M4**	**M5**	**M6**	**M7**	**M8**	**M9**
**Clinician- reported ECOG***		X														
**Symptomatic AE (PRO-CTCAE)**		X	X	X	X	X	X	X	X	X	X	X	X	X	X	X
**FACIT Item GP5**		X	X	X	X	X	X	X	X	X	X	X	X	X	X	X
**PGI-S**		X	X	X	X	X	X	X	X	X	X	X	X	X	X	X
**PROMIS Physical Function SF 8c**		X	X	X	X	X	X	X	X	X	X	X	X	X	X	X
**EORTC QLQ-F17 Role Function only**			X				X		X		X	X		X	X	
**PRO-ECOG**		X	X		X		X		X	X	X	X	X	X	X	X
**EORTC QLQ-F17**		X			X					X			X			X
**PGI-C**			X	X	X	X	X	X	X	X	X	X	X	X	X	X
**6MWT†**		X								X						
**Exit Questionnaire**																X
**Wearable Data**	X¥->	Continuous wearable data throughout														

BL – baseline, W - week, M – month;
ECOG – Eastern Cooperative Oncology Group
AE – Adverse event
PRO-CTCAE – Patient-Reported Outcomes version of the Common Terminology Criteria for Adverse Events
FACIT Item GP5 – Functional Assessment of Chronic Illness Therapy Item GP5
PGI-S – Patient Global Impression scale of severity
PROMIS Physical Function SF 8c – Patient-Reported Outcomes Measurement Information System Physical Function Short Form 8c
EORTC QLQ-F17 – European Organisation for Research and Treatment of Cancer Quality of Life Form 17
PRO-ECOG – Patient-Reported Outcomes version of the ECOG Performance Status
PGI-C – Patient Global Impression scale change
6MWT – 6-minute walk test
* at baseline (research assistant to ensure ECOG is recorded at baseline by clinical provider), and where available at follow up
** - context dependent long-term follow-up
† 6MWT at baseline and at M3 will be performed in clinic (with research assistant) for patients treated at primary sites available for assessment. The window for the M3 6MWT assessment is anytime during the 3rd month.
¥ Lead-in time period of at least 24 hours prior to initiation of cancer-directed treatment
Highlighted time points are "high yield" time points for reminders and will include research assistant phone calls to patient if Patient-Reported Outcomes have not been completed
