# Supplementary material for: Integrating 4 methods to evaluate physical function in patients with cancer (In4M): protocol for a prospective cohort study
Source: BMJ Open. 2024 Jan 10;14(1):e074030. doi: 10.1136/bmjopen-2023-074030 (PMC10806877; doi:10.1136/bmjopen-2023-074030)
Supplement: Supplementary data [file bmjopen-2023-074030supp002.pdf]

Appendix (Supplementary Material) 2: Exit Questionnaire

The In4M Study: Integrating 4 Methods to Assess Physical Function in Cancer Patients

Questions about your physical function (defined as the ability to carry out day to day activities that require physical effort)

1. How often did your cancer treatment affected your physical function? If you answer "Never" skip to question 4.

Never	Rarely	Occasionally	Frequently	Almost constantly

2. How much did your cancer treatment affected your physical function?

Not at all	A little bit	Somewhat	Quite a bit	Very much

3. How do you feel your physical function was affected over the course of your cancer treatment (open-ended)?

____

____

4. Compared to what you expected, how much did your cancer treatment affect your physical function?
   a. Cancer treatment affected my physical function less than I expected
   b. Cancer treatment affected by physical function about the same as I expected
   c. Cancer treatment affected my physical function more than I expected

5. What else would you have wanted to share with us about your physical function during this study that we did not ask (open-ended)?

____

____

Questions about the surveys you completed during this study

6. Did you feel that answering the questions asked on the Hugo platform was burdensome?
   a. Yes
   b. No
   c. Sometimes

7. Thinking about the way that your cancer treatments affected you in general, were there any other questions that you wish we had asked (open-ended)?

____

____

8. During this study, we asked you to choose a response for the statement of: "I am bothered by side effects of treatment." The response options ranged from "not at all" to "very much". When we asked you this question before you had started cancer treatment, how did you interpret it when answering? If you cannot recall, make your best guess.
   a. I answered as though it was asking me if I had any symptoms at that time
   b. I answered as though it was asking me about side effects of prior treatments for other medical conditions
   c. The question did not make sense to me since I have not previously received treatment for this cancer; I skipped the question
   d. The question did not make sense to me since I have not previously received treatment for this cancer; I chose "not at all" as my answer
   e. The question did not make sense to me since I have not previously received treatment for this cancer; I chose another response option besides "not at all" as my answer
   f. Other [please specify]:_______________________________________

____

____

9. When we asked you to choose a response for the same statement of: "I am bothered by side effects of treatment." during your cancer treatment, what factors did you consider when answering? (Select all that apply; multiple choices are allowed)
   a. The worst side effects I experienced (Severity)
   b. The most recent side effects I experienced (Recency)
   c. The most frequent side effects I experienced (Frequency)
   d. How long the side effects lasted (Duration)
   e. Any and all the side effects I had experienced to that point (Totality)
   f. Not applicable because I always answered "not at all" to this question
   g. Other [please specify]:_______________________________________

____

____

Questions about the wearable device (Fitbit)

10. Charging of the Fitbit was manageable.

Strongly disagree	Disagree	Neither agree nor disagree	Agree	Strongly Agree

11. Fitbit uploads were manageable.

Strongly disagree	Disagree	Neither agree nor disagree	Agree	Strongly Agree

12. How often did you check on your Fitbit to track your own activity?

Never	Once a month or less	Once a week	A few times a week	About once a day	Several times a day

If you chose "Never", skip to question 14.

13. If you did check on your Fitbit to track your own activity, how much did tracking your activity in real time with a wearable device (the Fitbit) influence your activity level?

Not at all	A little bit	Somewhat	Quite a bit

14. Do you think that using the Fitbit to track your activity could help your doctors and nurses to monitor your health and physical function, above and beyond using surveys only that ask about your health and physical function? Why or why not (open ended)?

15. Did you feel that the information from your Fitbit was an accurate reflection of your physical function? Why or why not (open ended)?

____

____

16. Was there anything about the Fitbit that bothered you during the study?

____

____

17. Is there anything else you would like to tell us about your participation in this study?

____

____
